# Lessons Learned From Limited Overlap of 15 In Vitro COVID-19 Drug Repurposing Screens

**DOI:** 10.1089/hs.2022.0132

**Published:** 2023-07-18

**Authors:** Phillip J. Tomezsko, Cassandra W. Phillipson, Matthew E. Walsh

**Affiliations:** Phillip J. Tomezsko, PhD, is Technical Staff, Counter WMD Systems Group, MIT Lincoln Laboratory, Lexington, MA.; Casandra W. Philipson, PhD, was Technical Staff, MIT Lincoln Laboratory, Lexington, MA.; Matthew E. Walsh was Associate Technical Staff, Biological and Chemical Technologies Group, MIT Lincoln Laboratory, Lexington, MA.; Matthew E. Walsh is currently a PhD Student, Department of Environmental Health and Engineering, Johns Hopkins Bloomberg School of Public Health, Baltimore, MD.

**Keywords:** COVID-19, Public health preparedness/response, Countermeasures, Biodefense R&D, Infectious diseases, High-throughput screen

## Abstract

Drug repurposing can quickly and cost-effectively identify medical countermeasures against pathogens with pandemic potential and could be used as a down-selection method for selecting US Food and Drug Administration-approved drugs to test in clinical trials. We compared results from 15 high-throughput in vitro screening efforts that tested approved and clinically evaluated drugs for activity against SARS-CoV-2 replication. From the 15 studies, 304 drugs were identified as displaying the highest level of confidence from the individual screens. Of those 304 drugs, 30 were identified in 2 or more screens, while only 3 drugs (apilimod, tetrandrine, and salinomycin) were identified in 4 screens. The lack of concordance in high-confidence hits and variations in protocols makes it challenging to use the collective data as down-selection criteria for identifying repurposing candidates to move into a clinical trial.

## Introduction

Pharmaceutical prophylactics and treatments are important medical countermeasures that can help prevent and respond to pandemics. However, upon the emergence of a novel pathogen, it is unconfirmed and unknown what drugs may be efficacious against that pathogen and associated disease. As evidenced by the COVID-19 pandemic, the time from identification of a pathogen with pandemic potential to the beginning of the pandemic can be just a few months. Therefore, to have the most impact, prophylactics and treatments need to be identified or developed quickly. Unfortunately, development of a novel drug is a long and expensive endeavor. The average time for a novel drug to advance from candidate discovery to US Food and Drug Administration (FDA) approval is 8.3 years, which does not include the highly variable basic research and development steps.^1^ The average cost of development for a new drug regularly exceeds US$1 billion. Given that clinically approved drugs represent the culmination of such large cost and time investments, repurposing approved drugs is an attractive avenue to find therapeutics for a novel pathogen or disease.^2^ Furthermore, approved drugs or those that have at least reached clinical evaluation generally have favorable safety, efficacy, and bioavailability profiles, as well as a known mechanism of action. As clinical trials have already been conducted on these drugs to characterize safety and bioavailability, using previously approved drugs can reduce time and cost in clinical trials for a new indication. In some cases, results that show safety and bioavailability from previous Phase 1 and 2 clinical trials can be leveraged to expedite the approval process. Hence, finding an efficacious repurposed drug has the potential to cut years off the drug development process and save countless lives during a pandemic.

Identifying repurposed drugs that have therapeutic effects on novel pathogens or diseases may be done using high-throughput screening (HTS) assays, which represent a popular strategy to rapidly test whether compounds exhibit promising activity against a target of interest. The most promising drugs are then moved into animal or clinical trials. Drugs not designed against specific targets of a novel pathogen are unlikely to yield highly potent hits.^3^ As such, there are limited examples of successful drug repurposing against a novel pathogen, and most successful drug repurposing efforts are serendipitous, anecdotal, or hypothesis driven.^2,3^ But even if only limited pathogen-targeting is demonstrated, repurposed drugs may interact with essential host cofactors and provide a near-term stopgap prophylaxis or treatment before more potent novel drugs can be developed. In response to the emergence of SARS-CoV-2, many research groups conducted and published HTS assays evaluating tens of thousands of drugs, hoping to find a safe and efficacious treatment of COVID-19. Collectively, these datasets represent a new opportunity to study the use of HTS assays for repurposing drugs against a pathogen with pandemic potential and to serve as the basis for recommendations for future preparedness measures.

In this analysis, we selected and compared 15 drug repurposing screening efforts for COVID-19 using susceptible mammalian cells and full-length SARS-CoV-2.^4-18^ We limited this analysis to screens that used full-length SARS-CoV-2 to include repurposed drugs that have either a direct or indirect effect on viral replication. Each of these studies confirmed the relevance of their hits. Most commonly, this involved screening the top hits in additional, physiologically relevant cell lines or using research compounds to confirm the target of the approved drug. Several studies tested hits using orthogonal technologies, including transcriptomic analysis and viral enzymatic activity assays. Using each author's own criteria for highest confidence for their candidates, we compared the top hits from each study. In total, these studies identified 304 high-confidence candidates, 18 of which were shared by 2 screens, 9 shared by 3 screens, and 3 shared by 4 screens. No inhibitor was common to 5 screens or more, except for remdesivir, which was included as a positive control in 13 studies.

## Methods

We conducted PubMed and Google Scholar searches using the following search terms:

**PubMed:** (“COVID-19”[mesh] OR “SARS-CoV-2”[mesh] OR “COVID-19”[tiab] OR “SARS-CoV-2”[tiab]) AND (“Repurposing”[tiab] OR “Repurposed”[tiab] OR “Repositioning”[tiab] OR “Repositioned”[tiab] OR “FDA-Approved”[tiab]) AND (“library”[tiab] OR “screen”[tiab] OR “high-throughput”[tiab] OR “large scale”[tiab] OR “screening”[tiab] OR “screened”[tiab])

**Google Scholar:** intitle:(“SARS-CoV-2” OR “COVID-19”) AND intitle:(“repurposing” OR “repositioning” OR “FDA-Approved”) AND intitle:(“screening” OR “high-throughput” OR “screen” OR “large scale”) site:biorxiv.org OR site:papers.ssrn.com OR site:researchsquare.com

The Google Scholar search was specifically designed to find studies from popular preprint servers that may not yet be available on PubMed. The 2 searches yielded a total of 764 entries: 413 entries from PubMed and 351 entries from Google Scholar. The searches were completed on March 18, 2021. After screening the studies for relevance by title and abstract, we excluded 353 and 292 papers from PubMed and Google Scholar, respectively, resulting in 119 papers in total. The most common reasons for exclusion in this step were the title and abstract made it clear their study was an *in silico*-only screen or that the abstract mentioned only a screen as motivation for the subsequent work.

After removing duplicates (such as a preprint and peer-reviewed version of the same paper), we conducted a full-text review of the remaining 101 studies. The following criteria were required for further inclusion. The study must have: completed an experimental drug screen in a cell model, used a library of at least 48 compounds, included FDA-approved compounds (even if the library also contained investigational compounds), and used full-length SARS-CoV-2. We determined 48 compounds to be a number representative of “high-throughput,” as multiples of 8 are often associated with microtiter plate formats, and we reasoned that 48 compounds would be overly burdensome to test on an individual basis. We considered cell-based assays only because we reasoned comparing cell-based assays and biochemical assays would not be expected to produce equivalent results. We also included the requirement for full-length SARS-CoV-2 to find drugs that could target any step of the viral replication cycle or an essential host cofactor. We chose to include investigational hits and FDA-approved compounds in our analysis to increase discovery of common drugs and pathways. Additionally, investigational drugs that have been evaluated in animal models and previous clinical trials are unlikely to undergo structure–activity relationship modifications, making them more similar to FDA-approved drugs than drug discovery leads. Any study failing to meet all inclusion criteria was excluded.

**Figure 1.**
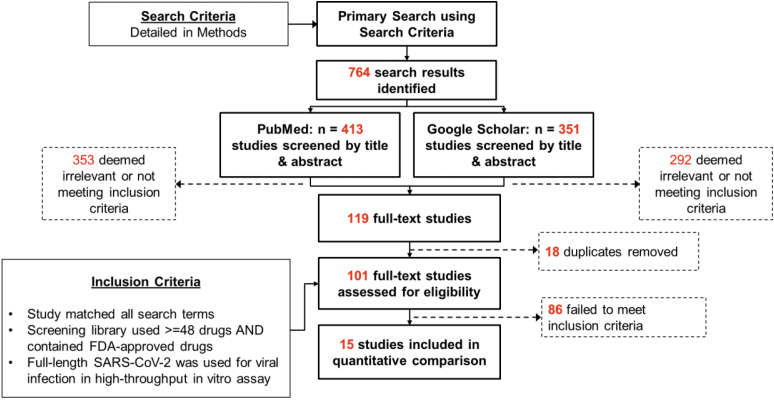
Search and inclusion criteria of studies used in this review. Abbreviation: FDA, US Food and Drug Administration.

We identified 15 publications that met all criteria for inclusion. The final list of high-confidence drug candidates were taken from the text of each publication. Drug names were standardized using Medical Subject Headings (ncbi.nlm.nih.gov/mesh/) to ensure that drug aliases did not confound the analysis. The shared hits were determined by merging the lists of standardized drug names. This down-selection process is summarized in Figure 1.

## Results

### Study Methodologies

We compared the results from a total of 15 independent screens. An overview of the different experimental conditions and follow-up experiments is found in Table 1. The number of drugs screened per study ranged from 48 to 12,000. The largest drug library used was the ReFRAME (Repurposing, Focused Rescue, and Accelerated Medchem) library, which is composed of 39% FDA-approved drugs, 58% investigational drugs, and 3% preclinical compounds.^4,13^ Twelve of these studies used Vero E6^5,7,9-11,13-18^ cells for screening, including 1 study using Vero E6 cells overexpressing ACE2.^6^ Other cell lines used included Huh7,^12^ Huh7.5,^7^ Caco-2,^8^ HeLa-ACE2,^4^ Calu-3,^5,7^ and human renal cortical epithelial cells.^9^ The most common assay readout was cytopathic effect of viral infection.^6,8,10,13,15-18^ Several studies used immunostaining of SARS-CoV-2 markers to quantify the number of infected cells.^4,7,11,14,18^ Two studies incorporated machine learning into the readout to detect changes in cell morphology.^9,12^

**Table 1.** Overview of Experimental Conditions and Confirmation Assays Carried Out by Each of the Studies Reviewed

Lead Author	Cell Line	Virus	Multiplicity of Infection	Readout	Compound Addition	Incubation Period	Confirmation Assay
Bakowski^4^	HeLa-ACE2	SARS-CoV-2 USA-WA1/2020	2.2	SARS-CoV-2 immunostaining	Concurrent with infection	24 hours	Synergy with remdesivir
Biering^5^	Vero E6; Calu-3	SARS-CoV-2 USA-WA1/2020	0.05	CPE	1 hour preinfection	72 hours; 96 hours	GSEA, viral load reduction assay; alternate cell lines, protease activity assays
Chen^6^	Vero-ACE2	SARS-CoV-2 USA-WA1/2020	0.002	CPE	Concurrent with infection	72 hours	NA
Dittmar^7^	Vero E6; Huh7.5	SARS-CoV-2 USA-WA1/2020	1.0	SARS-CoV-2 immunostaining	1 hour preinfection	30 hours	Validation in Calu-3, related compound testing for cyclosporine
Ellinger^8^	Caco-2	SARS-CoV-2 primary isolate	0.01	CPE	Concurrent with infection	48 hours	NA
Heiser^9^	Vero E6; HRCE	SARS-CoV-2 USA-WA1/2020	0.08; 0.4	Cell morphology	20 hours preinfection	48 hours	NA
Holwerda^10^	Vero E6	SARS-CoV-2 Muchen-1/2020/929	0.01	CPE	2 hour preinfection	48 hours	NA
Jan^17^	Vero E6	SARS-CoV-2 KOR/KCDC03/2020	0.5	CPE	24 hours preinfection	72 hours	Secondary screen
Jeon^11^	Vero E6	SARS-CoV-2 KOR/KCDC03/2020	0.0125	SARS-CoV-2 immunostaining	24 hours preinfection	24 hours	NA
Ku^16^	Vero E6	SARS-CoV-2 (hCoV-19/Taiwan/4/2020)	100 TCID_50_	CPE	Concurrent with infection	72 hours and 120 hours	Protease and RdRp activity assay; protease, RdRp, and E variant virtual screen; animal studies
Mirabelli^12^	Huh7	SARS-CoV-2 USA-WA1/2020	0.2	Cell morphology and SARS-CoV-2 immunostaining	4 hours preinfection	48 hours	Multi-cycle CPE reduction assay, synergy with remdesivir, validation in iAECs
Riva^13^	Vero E6	SARS-CoV-2 HKU-001a	0.01	SARS-CoV-2 immunostaining	16 hours preinfection	72 hours	Rescreen with immunoassay readout, transcriptomics, pseudovirus entry, validation in human cell lines and primary human cells, synergy with remdesivir
Sales-Medina^14^	Vero E6	SAR-CoV-2 SP02/human/2020/BRA	0.01	SARS-CoV-2 immunostaining	Concurrent with infection	48 hours	Network analysis
Touret^15^	Vero E6	SARS-CoV-2 BavPat1	0.002	CPE	15 minutes preinfection	72 hours	Validation in Caco-2 cells
Yuan^18^	Vero E6	SARS-CoV-2 HKU-001a	0.004	SARS-CoV-2 ELISA and CPE	1 hour postinfection	72 hours	Viral load reduction assay; plaque reduction assay; time of drug addition assay

Abbreviations: CPE, cytopathic effect; GSEA, Gene set enrichment analysis; HRCE, human renal cortical cells; iAECs, alveolar epithelial cells; RdRp, RNA-dependent RNA polymerase.

Each study defined the criteria for a high-confidence hit (Table 2). Eight studies conducted a primary screen followed by a more stringent secondary screen with additional criteria for hit ranking. The remaining 7 studies conducted a single screen and applied ranking criteria to that primary screen alone. Common thresholds were based on the half-maximal effective concentration (EC_50_) of the compounds, percentage reduction in cytopathic effect, and a selectivity index (SI) calculated by comparing the inhibition to a cytotoxicity counterscreen. In general, the high-confidence hits displayed a dose–response relationship with viral inhibition and had an EC_50_ < 10 μM.

**Table 2.** Criteria for Inclusion of a Drug Candidate as a High-Confidence Hit in Each of the Studies Reviewed

Lead Author	Primary Criterion 1	Primary Criterion 2	Secondary Criterion 1	Secondary Criterion 2	Secondary Criterion 3	Remdesivir EC_50_ (Cell Line)	Negative Control
Bakowski^4^	Reduced number of infected cells by 50%	No more than 40% of cells died	EC_50_ < 9.6 μM	Reconfirmed	CC_50_/EC_50_ > 10	0.194 μM (HeLa-ACE2)	Uninfected cells + DMSO
Biering^5^	>20% CPE inhibition in either Vero E6 or Calu-3	Favorable GSEA analysis	EC_50_ < 10 μM	CC_50_/EC_50_ > 10		2.45 μM (Calu-3; Vero E6)	Uninfected cells
Chen^6^	Reduced CPE efficacy by >55%		CPE efficacy >80%	EC_50_ < 10 μM	Selectivity index compared to counter screen >10	10 μM (Vero-ACE2)	Uninfected cells + DMSO
Dittmar^7^	<40% infected cells	>80% viability	EC_50_ < 10 μM in Vero E6, Calu-3, and Huh7.5	CC_50_/EC_50_ > 3 in Vero E6 and Huh7.5, >2 in Calu-3		0.002 μM (Huh7.5); 0.457 μM (Vero E6); 0.005 μM (Calu-3)	Uninfected cells + DMSO
Ellinger^8^	Inhibition efficacy >75		IC_50_ < 20 μM	IC_50_ < 1 μM		0.76 μM (Caco-2)	Uninfected cells
Heiser^9^	Hit score >0.6 of either dose in either cell line	Compound class with hit scores 0.45 by hypergeometric test with *P* < .05				0.95 score (HCRE); 0.59 (Vero E6)	Uninfected cells
Holwerda^10^	Reduced CPE	> -2 z-score in cell viability and cytotoxicity screen				0.17 μM (Vero E6)	Uninfected cells + DMSO
Jan^17^	Reduced CPE by 50%					Not tested	Uninfected cells + DMSO
Jeon^11^	EC_50_ < 10 μM					11.41 μM (Vero E6)	Uninfected cells + DMSO
Ku^16^	>60% CPE inhibition in 1 of 2 experiments					5 μM (Vero E6)	Uninfected cells
Mirabelli^12^	Morphology compared to positive and negative controls		Dose–response relationship	EC_50_ < 1 μM		0.018 μM (Huh7)	Uninfected cells
Riva^13^	>5 SD log2FC in LOPAC1280 screen	Top 100 hits in 1 of 2 ReFRAME screen replicates	Dose–response relationship	EC_50_ < 2.5 μM		0.54 μM (Vero E6)	Uninfected cells + DMSO
Sales-Medina^14^	EC_50_ > 1E-04 μM of μg/mL	CC_50_/EC_50_ > 18				Not tested	Uninfected cells + DMSO
Touret^15^	Inhibition index >1 (combines cell viability and comparison to 10 μM IC_50_ arbidol)					1.6 μM (Vero E6)	Uninfected cells
Yuan^18^	Reduced SARS-CoV-2 ELISA signal by >4-fold		>90% reduction in CPE			1.04 μM (Vero E6)	Uninfected cells + DMSO

Note: Remdesivir EC_50_ values and cell line tested are included, when available, as positive control measurements. Abbreviations: CPE, cytopathic effect; DMSO, dimethylsulfoxide; CC_50_, half-maximal cytotoxicity concentration; EC_50_, half-maximal effective concentration; ELISA, enzyme-linked immunosorbent assay; GSEA, gene set enrichment analysis; IC_50_, half-maximal inhibitory concentration.

### Study Results

**Figure 2.**
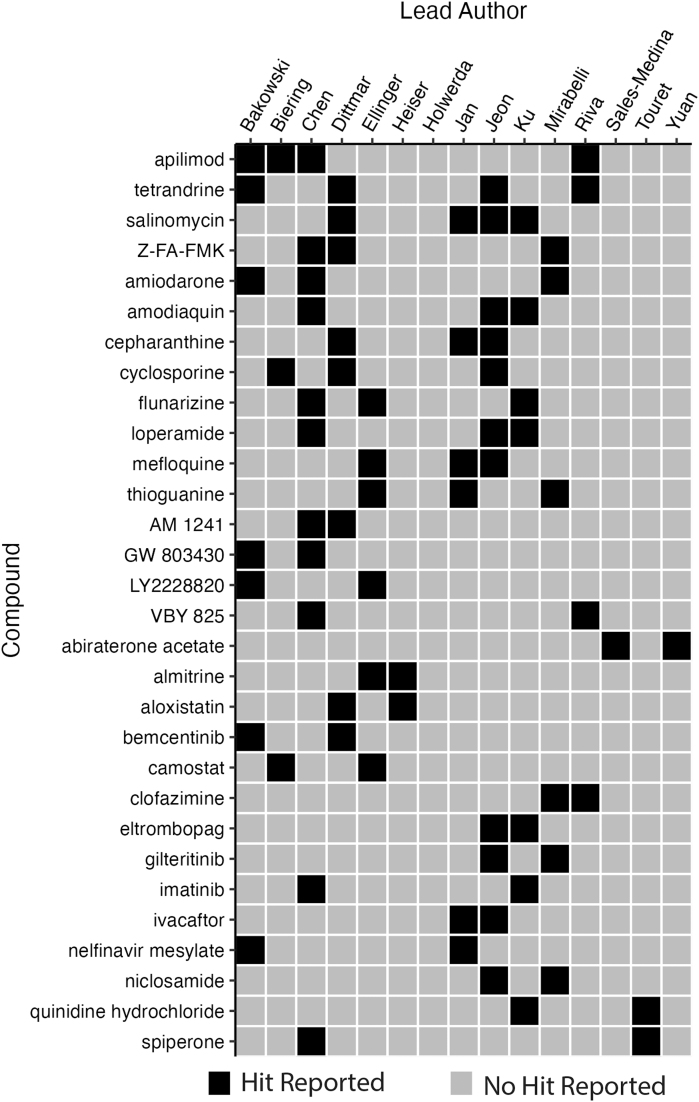
Binary heatmap of high-confidence reported hits shared by 2 or more studies.

**Figure 3.**
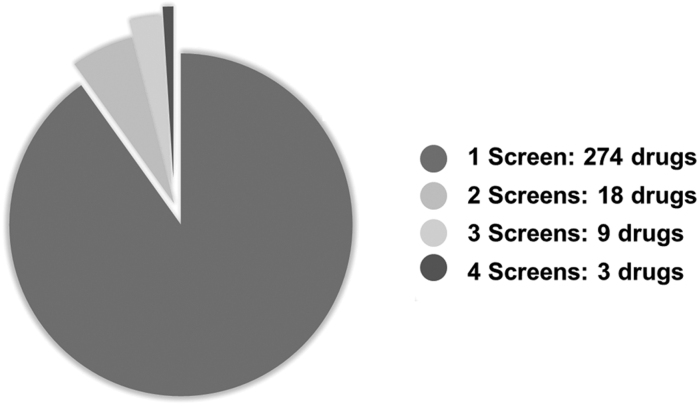
Pie chart of all compounds identified as high-confidence hits. A total of 274 high-confidence hits were uniquely identified by the study to identify each as a high-confidence hit.

**Figure 4.**
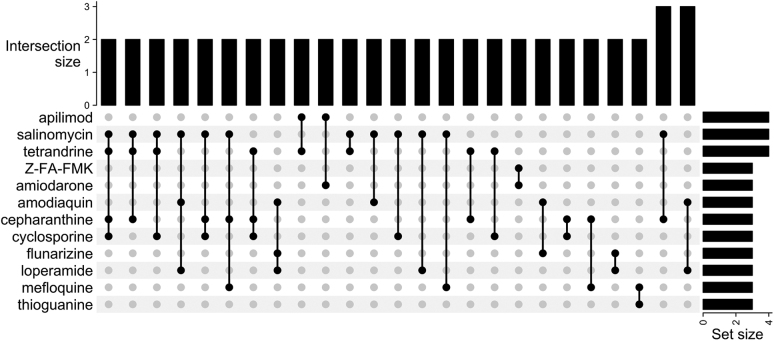
Upset plot of overlap among studies. The intersection size is defined as the number of studies that identified the corresponding combination of high-confidence hits. For example, 2 studies identified salinomycin, tetrandrine, cepharanthine, and cyclosporine as high-confidence hits. The set size is defined as the number of times a particular compound was defined as a high-confidence hit.

The screens collectively reported 304 high-confidence hits. Of these hits, 18 were shared by 2 screens, 9 were shared by 3 screens, and 3 (apilimod, salinomycin, and tetrandrine) were common to 4 screens (Figure 2). No high-confidence hit was common to 5 screens or more (Figure 3). The high-confidence hit rate from the assays ranged from 0.2% to 50.0% and is inversely correlated to library size. The study with a 50.0% hit rate was from the screen that tested 48 compounds,^11^ whereas the lowest hit rate came from 1 of the studies using the ReFRAME library (n=12,000 compounds).^13^ Of the hits from each study, the percentage of hits that were shared with at least 1 other study ranged from 0.0% to 45.8% (Figure 4, Table 3).

**Table 3.** Results and Characteristics of Each Study Reviewed

Lead Author	Cell Line(s)	*TMPRSS2 *Expression	Drugs Evaluated n	High-Confidence Hits n (%)	Hits Shared %
Bakowski^4^	HeLa-ACE2	No	12,000	25 (0.2)	28.0
Biering^5^	Calu-3; Vero E6	Yes; no	5,370	13 (0.2)	23.1
Chen^6^	Vero-ACE2	No	8,810	55 (0.6)	20.0
Dittmar^7^	Vero E6; Huh7.5; Calu-3	No; no; yes	3,059	23 (0.8)	34.8
Ellinger^8^	Caco-2	Yes	5,632	66 (1.2)	9.1
Heiser^9^	Vero-E6; HRCE	No; yes	1,670	21 (1.3)	9.5
Holwerda^10^	Vero E6	No	400	15 (3.8)	0.0
Jan^17^	Vero E6	No	2,855	15 (0.5)	40.0
Jeon^11^	Vero E6	No	48	24 (50.0)	45.8
Ku^16^	Vero E6	No	1,473	29 (2.0)	24.1
Mirabelli^12^	Huh7	No	1,423	16 (1.1)	37.5
Riva^13^	Vero E6	No	12,000	20 (0.2)	20.0
Sales-Medina^14^	Vero E6	No	65	4 (6.2)	25.0
Touret^15^	Vero E6	No	1,520	15 (1.0)	13.3
Yuan^18^	Vero E6	No	1,528	4 (0.3)	25.0

Notes: Cell lines(s) include those used in primary and confirmation screening assays. TMPRSS2 Expression denotes if the corresponding cell line expresses the transmembrane serine protease 2. The percentage of high-confidence hits is defined as the number of high-confidence hits divided by the number of drugs evaluated multiplied by 100. The percentage of hits shared is defined as the number of high-confidence hits from a that study identified as high-confidence hits from at least 1 other study divided by the number of high-confidence hits in that study, multiplied by 100. Abbreviations: HRCE, human renal cortical cells; TMPRSS2, transmembrane serine protease 2.

Remdesivir was included either in the compound libraries or as a positive control in 13 of 15 studies (Table 2). The dose response to remdesivir was used as an indicator of the consistency between assays. The measured EC_50_ of remdesivir ranged from 0.002 μM to 11.41 μM. Specifically, remdesivir yielded about a 70-fold range in EC_50_ in Vero E6 cells (0.17 μM to 11.41 μM) and about a 600-fold range in the 2 studies that used Calu-3 cells (0.005 μM to 2.45 μM).^5,7^ Similar to remdesivir, apilimod showed cell-type dependent effects that had a roughly 1,000-fold range. In Vero E6 and HeLa-ACE2 cells, apilimod showed a robust EC_50_ of 0.023 μM to 0.050 μM. In Calu-3 cells, apilimod retained some activity at 4.54 μM.^7^ Given the large range of EC_50_ values, it is clear that cell type alone does not account for all the variability in measurements among HTS assays. Other likely experimental variables that contributed to the variability between screens are the time of compound addition, choice of output, and analysis method. These findings highlight the fact that experimental design variables beyond cell type have a large impact on assay output.

## Discussion and Recommendations

To facilitate a straightforward comparison of studies and to maximize the combined value of published datasets, future HTS work should:

**Clearly describe rationale and/or optimization of multiplicity of infection and compound addition protocol.** Important differences in experimental design were choices of cell line, time of compound addition, multiplicity of infection, hit inclusion criteria, and experimental output. The full array of protocols and experiments that the studies used to verify hits is in Table 1. Several of the works reviewed used multiple cell lines and meaningfully incorporated discordant results in the cell lines into their conclusions. While the rationale behind the choice of cell line was typically explained, the rationale behind the time of compound addition relative to infection and multiplicity of infection was almost never explained, and no optimization of those steps was shown. The time of drug treatment relative to infection could have a large impact on the types of inhibitors that can be identified. For example, pretreatment with inhibitors for 12 hours or more allows enough time for cells to make transcriptional and translational changes, influencing the ability to detect indirect effects. These effects may not be seen when a drug is added concurrently with infection. The many differences in protocols make it challenging to identify a hierarchy among conflicting HTS results and burden down-selection efforts of advancing drugs to clinical trials.

**Provide standardized drug names for all compounds tested and make screening and normalization data available with the publication**. In this analysis, it was not possible to ascertain the negative results for all studies. Some studies did not release the full dataset, while others were still in the preprint phase and may or may not have released the full dataset upon publication. (Between the time of our initial literature search and publication of this manuscript, 3 preprints were peer-reviewed and published but did not include additional data.^5,6,12^) As such, it is often not possible to know if a drug was a negative hit or not tested. However, discrepancies in the drug list cannot entirely explain the limit in overlap. For example, 2 studies used the extensive approximately 12,000 compound ReFRAME library but shared just 2 top candidates.^4,13^ Furthermore, the difference in outputs makes a direct comparison difficult even with the complete datasets. The high-content imaging machine-learning approaches are not easily amenable to compare against cytopathic effect reduction assays. Similarly, screens that used a multitiered approach to down-select compounds make direct comparisons difficult.

**Measure and report the selectivity index of all compounds tested.** In addition to having a low EC_50_, an ideal repurposing candidate should have a high SI, which indicates that it has antiviral effects beyond cytotoxicity. Apilimod and salinomycin had an SI of about 100 or higher, whereas tetrandrine displayed cytotoxicity and had an SI as low as 5. However, not all studies equally employed the use of SI within their hit inclusion criteria. Studies that did not consider SI when determining a hit were more likely to identify cytotoxic drugs at hits.

### Limitations of Analysis

Despite the relatively low overlap of potential inhibitors, 30 drugs were shared between at least 2 screens, and some of these drugs showed comparable or better potency to remdesivir. A highly potent inhibitor would be expected to be robust enough to show activity regardless of slight differences in protocol. However, our comparison identified only 3 consistent positive hits across 15 independent studies. It is difficult to tell if this seeming lack of concordance in results is due to bona fide biological effects or because very few of the compounds have strong activity against SARS-CoV-2.

The current analysis does not take into consideration that several of the drugs identified are derivatives of one another or belong to the same class of compound. A more detailed accounting of this may provide more clarity on the relationships among screens. However, when the cost to empirically measure candidates is low, there is some merit in not overclustering classes, as differences in specificity and even related targets can have large effects on the mechanism of action. Some studies found multiple hits for the derivatives or the same class of compound. For example, Heiser et al^9^ listed sirolimus and 3 closely related derivatives—zotarolimus, everolimus, and temsirolimus—as top hits. This indicates that in their system, the mechanistic target of rapamycin (mTOR) pathway is an especially important target, more so than other pathways with a single high-confidence hit.

## Conclusion

HTS efforts hold promise to find drugs that could be repurposed and quickly moved into clinical trials to evaluate their efficacy in preventing infection and death. A balance of exploration of new methods and robust, reproducible assays is desired. Exploration is important because many aspects of pathogen biology are unknown, and sticking to rigid protocols may risk missing potential therapeutics. However, rigid protocols facilitate straightforward comparisons across studies. Due to the high frequency of protocol variations, none of the 15 studies that we included in this work were an exact replicate of any other. Alternatively, it is possible that this discordance results from there being few drugs among the approved and clinically evaluated libraries with high antiviral activity against SARS-CoV-2. Ultimately, the incompatibility of the datasets renders the HTS assays an ineffective way to down-select drug repurposing candidates. Future pandemic preparedness and response efforts should consider this when designing experimental systems.
